# Genome-Wide Expression Profiling Identifies Type 1 Interferon Response Pathways in Active Tuberculosis

**DOI:** 10.1371/journal.pone.0045839

**Published:** 2012-09-21

**Authors:** Tom H. M. Ottenhoff, Ranjeeta Hari Dass, Ninghan Yang, Mingzi M. Zhang, Hazel E. E. Wong, Edhyana Sahiratmadja, Chiea Chuen Khor, Bachti Alisjahbana, Reinout van Crevel, Sangkot Marzuki, Mark Seielstad, Esther van de Vosse, Martin L. Hibberd

**Affiliations:** 1 Infectious Disease, Genome Institute of Singapore, Singapore, Singapore; 2 Department of Infectious Diseases, Leiden University Medical Center, Leiden, The Netherlands; 3 Eijkman Institute for Molecular Biology, Jakarta, Indonesia; 4 Health Research Unit, Faculty of Medicine, Padjadjaran University, Hasan Sadikin Hospital, Bandung, Indonesia; 5 Department of Internal Medicine, Radboud University Nijmegen Medical Center, Nijmegen, The Netherlands; National Institute for Infectious Diseases (L. Spallanzani), Italy

## Abstract

Tuberculosis (TB), caused by *Mycobacterium tuberculosis* (*M.tb*), remains the leading cause of mortality from a single infectious agent. Each year around 9 million individuals newly develop active TB disease, and over 2 billion individuals are latently infected with *M.tb* worldwide, thus being at risk of developing TB reactivation disease later in life. The underlying mechanisms and pathways of protection against TB in humans, as well as the dynamics of the host response to *M.tb* infection, are incompletely understood. We carried out whole-genome expression profiling on a cohort of TB patients longitudinally sampled along 3 time-points: during active infection, during treatment, and after completion of curative treatment. We identified molecular signatures involving the upregulation of type-1 interferon (α/β) mediated signaling and chronic inflammation during active TB disease in an Indonesian population, in line with results from two recent studies in ethnically and epidemiologically different populations in Europe and South Africa. Expression profiles were captured in neutrophil-depleted blood samples, indicating a major contribution of lymphocytes and myeloid cells. Expression of type-1 interferon (α/β) genes mediated was also upregulated in the lungs of *M.tb* infected mice and in infected human macrophages. In patients, the regulated gene expression-signature normalized during treatment, including the type-1 interferon mediated signaling and a concurrent opposite regulation of interferon-gamma. Further analysis revealed *IL15RA*, *UBE2L6* and *GBP4* as molecules involved in the type-I interferon response in all three experimental models. Our data is highly suggestive that the innate immune type-I interferon signaling cascade could be used as a quantitative tool for monitoring active TB disease, and provide evidence that components of the patient’s blood gene expression signature bear similarities to the pulmonary and macrophage response to mycobacterial infection.

## Introduction

Approximately one third of the world’s population is estimated to be latently infected with *Mycobacterium tuberculosis* (*M.tb*), the etiological agent of tuberculosis (TB), which is responsible for about 8–10 million active new cases of TB each year [1], [2]. A bacteriologically confirmed diagnosis of TB is challenging, as direct microscopy has low sensitivity, bacterial culture takes 2 to 4 weeks, and sputum samples can be microbiologically false negative. Effective treatment of active TB is crucial in containing spread of the disease, and better diagnostics would greatly impact on TB control by facilitating early treatment. Little is known about the precise mechanisms and pathways that control protective versus pathogenic immunity during TB [3], [4]. The IL-12/IFN-γ and the TNF/TNFR axis are both crucial in protection during *M.tb* infection, as genetic (*e.g.* defects in the IL-12/IL-23/IFN-γ/STAT1 axis) or acquired (HIV; immune-suppressants; anti-cytokine auto-antibodies) defects strongly increase host-susceptibility to mycobacterial disease [5]–[7]. IFN-γ is produced mostly by CD4+ T cells and assists macrophages in controlling intracellular *M.tb* infection through various anti-microbial immunity pathways, which include phagosomal maturation; anti-microbial peptides; oxidative stress; apoptosis and autophagy [8]. Although IFN-γ is essential to protection against *M.tb*, it is not in itself a correlate of protection, however.

Several studies have profiled gene expression patterns in peripheral blood from TB patients [9]–[15]. In a most comprehensive recent study, Berry *et al.* [15] reported a previously unappreciated role of IFN-αβ signaling in TB pathogenesis. Blood transcriptional profiles of active TB patients were dominated by a neutrophil driven IFN-αβ inducible gene expression profile [15]. A more recent study compared gene expression patterns in the blood of tuberculosis and sarcoidosis patients (of European descent), a chronic inflammatory lung disease with granulomatous inflammatory pathology similar to TB, and observed a very similar pattern in gene-expression, miRNA expression and serum cytokine levels between the two diseases [16]. These data strongly suggest the presence of a major core pro-inflammatory pathway in TB and sarcoidosis, although also disease specific patterns could be distinguished marking TB and sarcoidosis. Thus there has been substantial success in identifying biomarkers associated with active TB.

Notwithstanding the value of these two genome wide gene expression studies reported recently, a major outstanding question is whether these signatures, captured mostly in EU and South African populations, also translate to populations and regions with a very different host genetic and TB epidemiological background. Our study addresses this issue directly by assessing unbiased whole genome expression profiles in Indonesia, an Asian country with the third highest TB prevalence worldwide. Specifically, we have examined gene expression patterns in PBMC from a longitudinal patient cohort during active TB, TB treatment and TB convalescence. Moreover, we replicated the findings in two independent model systems, a mouse *M.tb*-infection model and a mycobacterium-infected human macrophage cell line model. Although mouse models typically do not develop caseous necrotic lesions, the hallmark of human pulmonary TB [17], both mice and humans develop TB granulomas in the lung with activation of similar pro-inflammatory and anti-inflammatory responses [18], and develop highly parallel innate and adaptive immune responses during *M.tb* infection [8], [19], [20]. Our results validate and significantly extend the results available in the current literature, extend these to different ethnic and TB epidemiological settings, and further refine the pro-inflammatory biomarker signature associated with active TB.

## Results

### Gene Expression Profiles of Active TB Patients Versus Healthy Controls in a Longitudinal Patient Cohort from Indonesia

**Figure 1:**
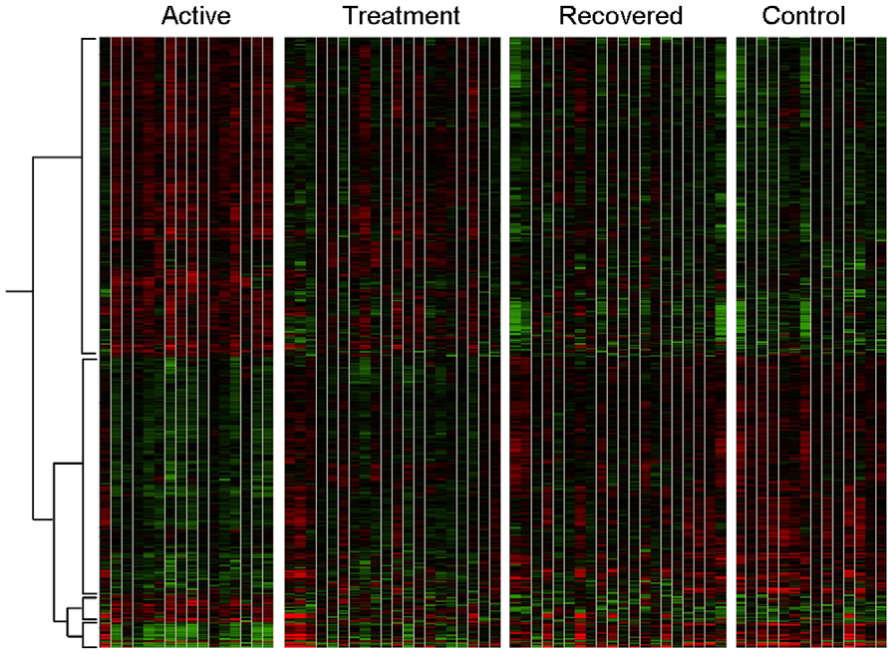
Differential gene expression in blood from TB patients and controls determined by microarrays. Heat map of 875 transcripts which were statistically different in one condition from the other condition, using ANOVA and were hierarchically clustered. active = patients with active disease, treatment = patients after 8 weeks of treatment, recovered = patients after 28 weeks of treatment and controls = healthy controls. Red represents an increase in gene expression and green represents a decrease in gene expression.

To identify molecular signatures involved in active TB, we applied microarray analysis to compare global gene expression in 23 patients with active TB (t = 0 weeks), whilst they were undergoing treatment (t = 8 weeks), and after they had completed the entire course of treatment (t = 28 weeks). The same microarray analysis was also applied to 23 matched healthy controls from the same population from the same area (see Methods). A total of 875 transcripts were significantly differentially expressed with an additional filter of a fold change greater than 2 compared to controls. The heatmap generated by these transcripts is shown (**Figure 1**). Looking at the overall gene-expression profile patterns as a continuum from active disease (t = 0 weeks) to treatment (t = 8 weeks) and cure (t = 28 weeks), we observed that global gene expression profile patterns were more similar for patients in the active disease stage (t = 0 weeks) and at the treatment stage (t = 8 weeks), whilst the gene expression profile patterns for patients after 28 weeks of curative treatment were more similar to those of the healthy controls.

### Active TB Disease Involves the Expression of Genes Related to Several Branches of Immunity

Gene ontology clustering was performed on the 875 differentially expressed transcripts described above using the Panther analysis software, comparing them to the entire reference list (N ≥24,000) of genes. Of the 875 transcripts 460 fall within eight broad groupings which were most statistically significant: amongst others immunity and defense, interferon-mediated and macrophage-mediated immunity, and the chemokine and cytokine pathways (**Table 1**, Bonferroni-corrected *P*-values <0.05 for all the groups).

**Table 1:** The distinct pathways in which gene ontology clusters 460 of the 875 differentially expressed genes.

Biological process	Total numberof genes	Number of genes differentially expressed between active TB patients and controls	Expected numberof genes	Bonferroni correctedp-value
Immunity and defense	1393	126	53.85	3.57×10^−17^
Interferon mediated immunity	69	17	2.67	5.15×10^−7^
Macrophage-mediated immunity	150	24	5.8	1.57×10^−6^
Cytokine/chemokine mediated signaling pathway	272	31	10.51	3.53×10^−5^
Natural Killer Cell mediated immunity	77	14	2.98	4.21×10^−4^
Apoptosis	606	44	23.42	2.2×10^−3^
T-cell mediated immunity	210	21	8.12	1.5×10^−2^
Signal transduction	3791	183	146.54	2.1×10^−2^

Significant pathways and biological processes overrepresented in differentially expressed genes between actively diagnosed patients and healthy controls from Panther analysis. 460 out of the 875 transcripts identified clustered into significant biological pathways and processes. A Bonferroni-corrected *P*<0.05 was considered statistically significant.

### Differential Gene Regulation in Humans During Active TB Disease and Upon Treatment

**Figure 2:**
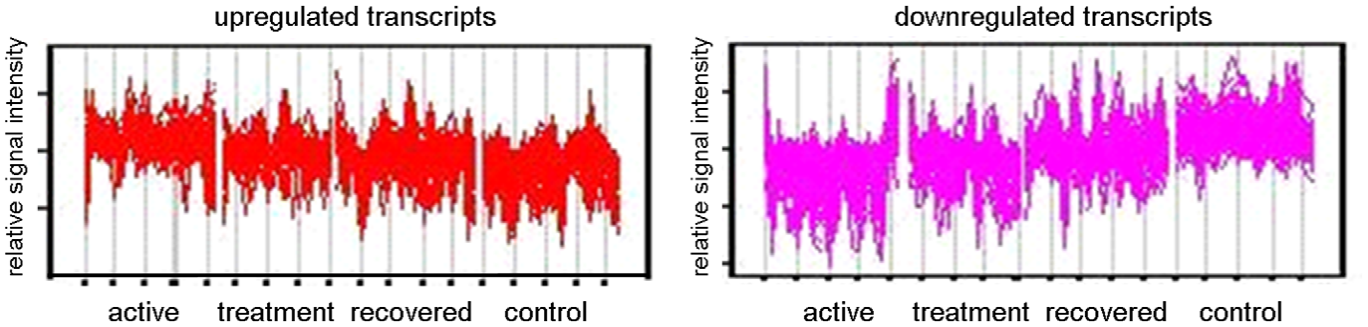
Cluster analysis using self-organizing map with further Panther analysis. Genespring GX10 was used to analyse transcripts which were significantly different between the four conditions (Active disease, Treatment, Recovered and Controls) using one-way ANOVA and subsequently subjected to self-organizing map clustering analysis. Upregulated transcripts (left panel) and downregulated transcripts (right panel) were subjected to further analysis through Panther.

Further analysis was undertaken by hierarchically clustering the 875-transcript list using the Self Organizing Map (SOM) algorithm in Genespring GX10, taking into account the patients’ disease stage at the time of sampling (untreated active disease (t = 0), undergoing treatment (t = 8 weeks), during convalescence after curative treatment (t = 28 weeks)) and for comparison, healthy controls. In order to distinguish between different expression profiles across these four conditions, ANOVA was used and the 875 statistically significant transcripts discovered from gene ontology clustering were initially subjected to a 3 row by 4 column SOM matrix (model free). Transcripts which exhibited the same behavior were clustered together, and two groups of transcripts (**Figure 2**) can be observed to have the clearest evidence of increased (**Figure 2**, left panel) or decreased expression (**Figure 2**, right panel) when comparing with active TB (t = 0 weeks), with time points during treatment (t = 8, 28 weeks), as well as comparing with controls. Both clusters were investigated for gene ontology clustering using Panther analysis. Analysis with the group of transcripts showing significantly increased expression revealed 56 genes which were significantly involved in discrete biological pathways. The type 1 interferon mediated immunity (a subgroup of immunity and defense), underlined by interferon α/β-mediated signaling, was highlighted as a very significant biological sub-process, (*P* = 5.21×10^−8^; **Table 2**). Conversely, analysis with the group of transcripts showing significantly decreased expression revealed 88 genes involved in significant biological pathways. Apart from immunity and defense (*P* = 5.84×10^−11^), other highly significant biological processes highlighted include chemokine and chemokine-mediated signaling (*P* = 4.53×10^−5^ **Table 3**). Interestingly, a concurrent down-regulation of interferon-gamma was observed.

**Table 2:** The distinct pathways in which gene ontology clusters 56 of the genes with increased expression normalizing during treatment.

Biological process	Total numberof genes	Number of genes with significantlyincreased expression in active TB patientsversus controls, normalizing duringTB treatment	Expected numberof genes	Bonferroni correctedp-value
Immunity and defense	1393	39	11.20	1.83×10^−10^
Type-I interferon mediated immunity	69	10	0.55	5.21×10^−8^
Macrophage mediated immunity	150	7	1.21	0.034

Significant Panther biological processes was detected for 56 up-regulated genes, type 1 interferon expression is increased in this group.

**Table 3:** The distinct pathways in which gene ontology clusters 88 of the genes with decreased expression normalizing during treatment.

Biological process	Total numberof genes	Number of genes with significantlydecreased expression in active TBpatients versus controls normalizingduring TB treatment	Expected numberof genes	Bonferroni correctedp-value
Immunity and defense	1393	35	8.93	5.84×10^−11^
Cytokine and chemokine mediated signaling pathway	272	12	1.74	4.53×10^−5^
Ligand mediated signaling	451	14	2.89	2.83×10^−4^
Cytokine/chemokine mediated immunity	123	7	0.79	2.42×10^−3^
Apoptosis	606	13	3.88	4.76×10^−3^
Macrophage mediated immunity	150	7	0.96	8.42×10^−3^

Significant Panther biological processes were detected with 88 down-regulated genes; type 2 interferon expression is decreased in this group.

### Comparison of Gene Expression Signatures from the Longitudinal TB Cohort Versus Those Obtained in a Human *in vitro* Macrophage Infection Model

**Figure 3:**
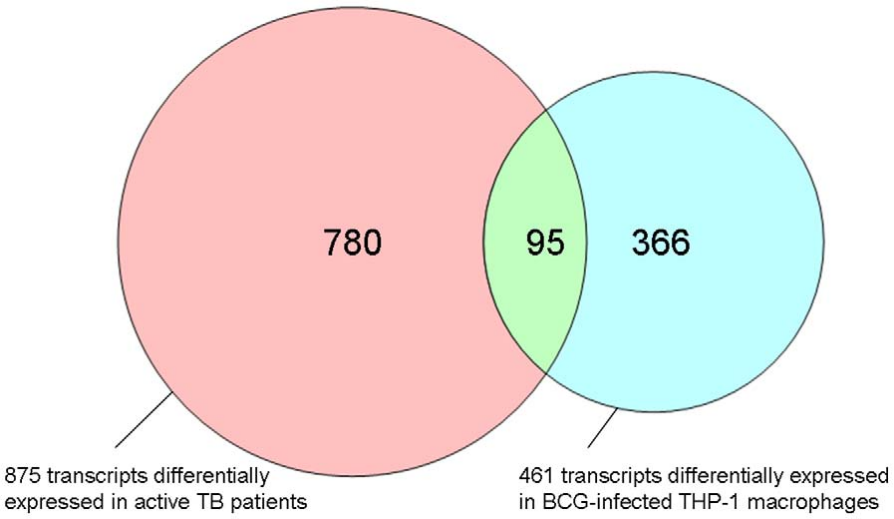
Comparison of differential gene expression between *M.tb* infected patients and BCG infected THP-1 cells. Venn diagram representing the 875 significantly differentially expressed in TB patients and 461 transcripts significantly differentially expressed in THP-1 BCG *in vitro* model. A total of 95 transcripts were found to be in common between these two systems. Red – transcripts for active TB patients only; Green – Common between TB patients and BCG-infected THP-1 cell line; Blue – transcripts for BCG-infected THP-1 only.

In order to examine whether the findings using patient blood mRNA responses could be confirmed in a well defined *in vitro* human macrophage (THP-1) cell line infection model, THP-1 cells were infected with the TB vaccine strain, *M.bovis* BCG (for 20 hours). A total of 461 transcripts were found by microarray analysis to be significantly differentially expressed in THP-1 macrophages following infection with BCG compared to uninfected control cells. A significant proportion, 95 out of these 461 transcripts, was found to be shared with the significantly differentially *in vivo* expressed transcripts in the above patient cohort (**Figure 3**). These 95 transcripts were investigated for functional clustering using Panther analysis, of which 77 out of the 95 were found to be significantly involved within functional clusters. The most significant biological process was again found to be in interferon mediated immunity (*P* = 1.14×10^−17^; **Table 4**).

**Table 4:** Differentially expressed genes in common between active TB patients versus controls and BCG-infected versus uninfected THP-1 cells.

Biological process	Total numberof genes	Number of genes in common betweenactive TB patients vs controls andBCG-infected vs uninfected THP-1 cells	Expected numberof genes	Bonferroni correctedp-value
Interferon-mediated immunity	69	14	0.29	1.14×10^−17^
Immunity and defense	1393	31	5.8	9.22×10^−14^
Cytokine/chemokine mediated signaling pathway	272	10	1.13	4.43×10^−5^
Cytokine/chemokine mediated immunity	123	7	0.51	1.37×10^−4^
Macrophage mediated immunity	150	6	0.62	6.08×10^−3^
Ligand-mediated signaling	451	9	1.88	2.26×10^−2^

Significant Panther biological processes were detected from 77 out of the 95 over-represented genes common between the two models.

### Human Host Responses to *M.tb* Infection Common with an *in vivo* Mouse Infection Model

**Figure 4:**
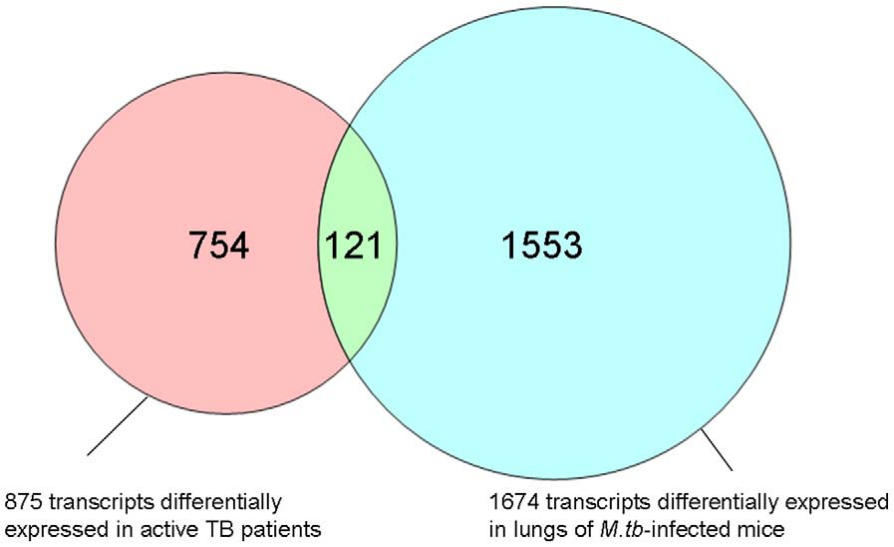
Comparison of differential gene expression between patients with active TB and mice infected *M.tb in vivo* model. Venn diagram representing the 875 significantly differentially expressed in TB patients and 1674 transcripts significantly differentially expressed in TB actively infected mice. A total of 121 transcripts were found to be in common between these two systems. Red – transcripts for TB patient only; Green – Common between TB patients and mice infected with TB; Blue – transcripts for mice infected with TB only.

In a third independent approach, we used an *in vivo* mouse TB infection model to examine whether the findings in the longitudinal patient cohorts blood samples could be confirmed in the infected mouse lung, the major organ affected by TB. A total of 1,674 transcripts were found by microarray analysis to be significantly differentially expressed in C57BL/6 mice infected with *M.tb* compared to non-infected controls. Of these transcripts, 121 were common to the significantly differentially expressed transcripts in the human cohort (**Figure 4**). These common transcripts were subjected to Panther gene ontology analysis, and 85 out of the 121 transcripts clustered into significant biological pathways. The most significant biological pathway was once again type1 interferon-mediated immunity (**Table 5**).

**Table 5:** Differentially expressed genes in common between active TB patients versus controls and TB-infected versus uninfected mice.

Biological process	Total numberof genes	Number of genes in common betweenactive TB patients vs controls andTB-infected vs uninfected mice	Expected numberof genes	Bonferroni correctedp-value
Immunity and defense	1393	39	7.46	6.71×10^−17^
Interferon-mediated immunity	69	12	0.37	7.90×10^−13^
Macrophage mediated immunity	150	11	0.8	1.02×10^−7^
Cytokine/chemokine mediated signaling pathway	272	11	1.46	5.63×10^−5^
Cytokine/chemokine mediated immunity	123	7	0.66	7.48×10^−4^
Natural Killer cell mediated immunity	77	5	0.41	9.51×10^−3^

Significant Panther biological processes were detected from 85 out of the 121 over-represented genes common between the two models.

### Genes Found in Common in All 3 Models of TB

**Figure 5:**
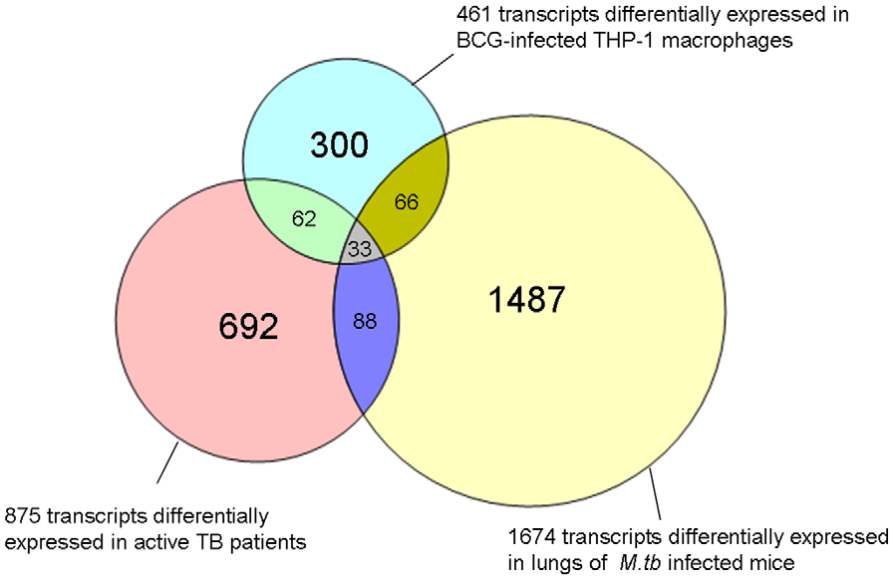
Comparison of differential gene expression from all three models. Venn diagram representing the 875 significantly differentially expressed in TB patients, 461 significantly differentially expressed in BCG-infected THP-1 cells and 1674 transcripts significantly differentially expressed in TB actively infected mice. A total of 33 transcripts (representing 26 genes) were found to be in common between all three systems. Red – transcripts for patients with active TB; Blue – transcripts for BCG-infected THP-1 cells; yYellow – transcripts for mice infected with active TB.

A total of 26 genes (or 33 entities due to the presence of multiple transcripts for several genes) were found to be significantly differentially expressed in all 3 models (longitudinal human TB cohort, human macrophage cell line, and *in vivo* mouse model; **Table 6** **,** **Figure 5**). These 26 genes were then subjected to Ingenuity Pathway Analysis (IPA). The most significant network (*P*<10^−32^) includes 18 focus molecules showing a high level of interaction between these molecules which involves both NFκB activation and the type I interferon response.

**Table 6:** Validation by qRT-PCR of 26 genes differentially expressed in all three models based on microarray analyses.

gene	Validated in longitudinal cohort TB patients	Validated in BCG-infected human macrophages	Validated in *M.tb*-infectedlungs of mice	Direction of expression change^a^
BATF2	yes	yes	no	up
C1QC	yes	no	yes	up
CCL2	no	no	no	down
CCL7	no	no	no	down
CD40	yes	yes	no	up
CX3CR1	yes	no	yes	up
CXCL10	no	no	no	up
DDX58	yes	yes	no	up
GBP4	yes	yes	yes	up
IFIT2	no	no	no	up
IFIT3	no	no	no	up
IL15RA	yes	yes	yes	up
IL1A	no	no	no	Down
IMPA2	yes	yes	no	Up
MEFV	yes	yes	no	Up
OAS1	yes	yes	no	Up
OAS2	yes	yes	no	Up
PBEF1	no	no	no	Down
PIM1	yes	yes	no	Up
SLAMF7	no	no	no	Up
TLR5	yes	yes	no	Up
TNFAIP2	yes	yes	no	Up
TRAFD1	yes	yes	no	Up
TRIM5	yes	yes	no	Up
UBE2L6	yes	yes	yes	Up
WARS	no	no	no	Up
	17 genes	15 genes	5 genes	

For successful validation, the direction for the fold-change in gene expression using qRT-PCR should be consistent with the microarray findings.

a in longitudinal cohort of TB patients.

**Figure 6:**
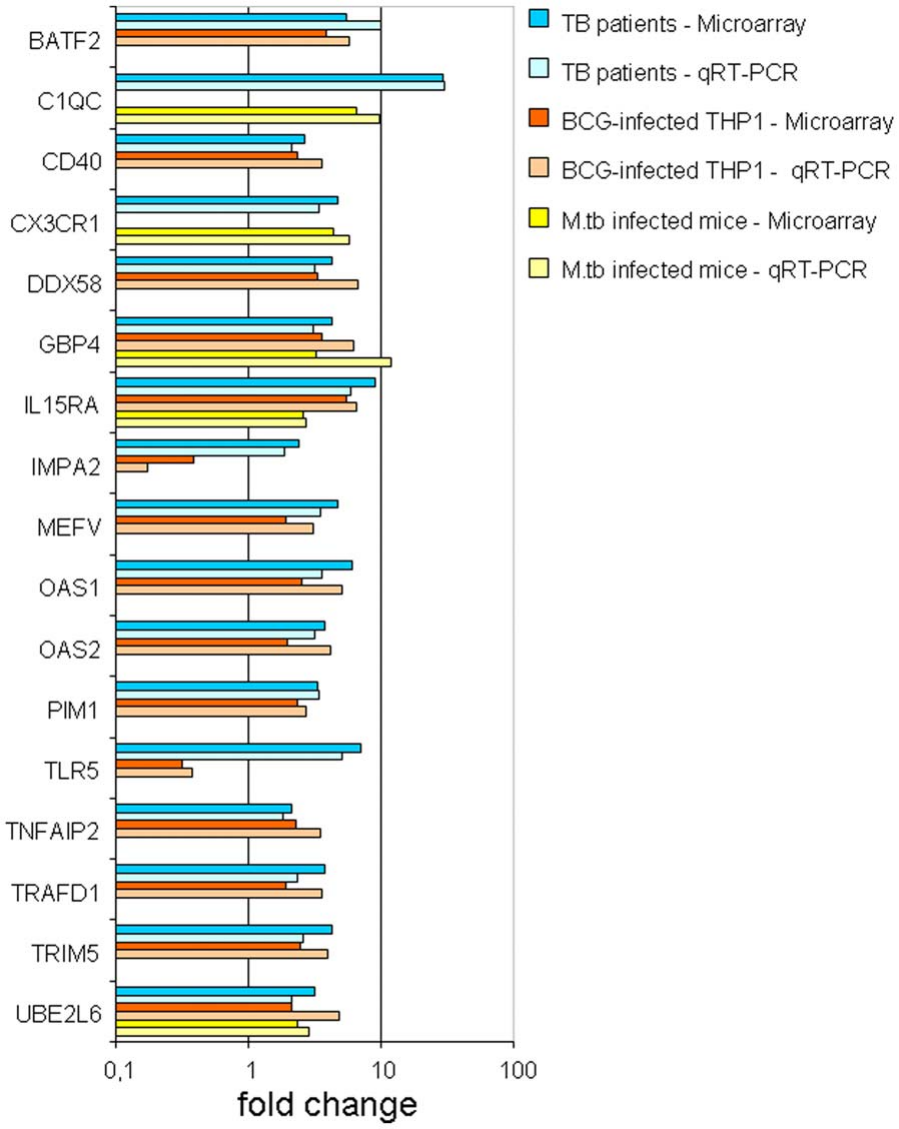
Validating microarray data by qRT-PCR. Microarray results were validated by qRT-PCR. 17 genes from the 26 genes were found to be significantly different between TB patients with active disease and healthy controls, 15 genes from the 26 genes were found to be significantly different between live BCG-infected THP-1 cells at 20 hours compared to uninfected controls, and 5 genes from 26 genes were found to be significantly different between mice infected with active TB and mock infected mice, using student’s T test (P<0.05). Three genes which were common to all 3 models show similar levels of expression with both techniques applied. The fold increase for microarray technique (blue, orange and yellow) and qRT-PCR technique (light blue, light orange and light yellow) for patients, THP-1 cells and mice respectively. Microarray data (and their corresponding qRT-PCR data) that were not validated by qRT-PCR are not shown.

**Figure 7:**
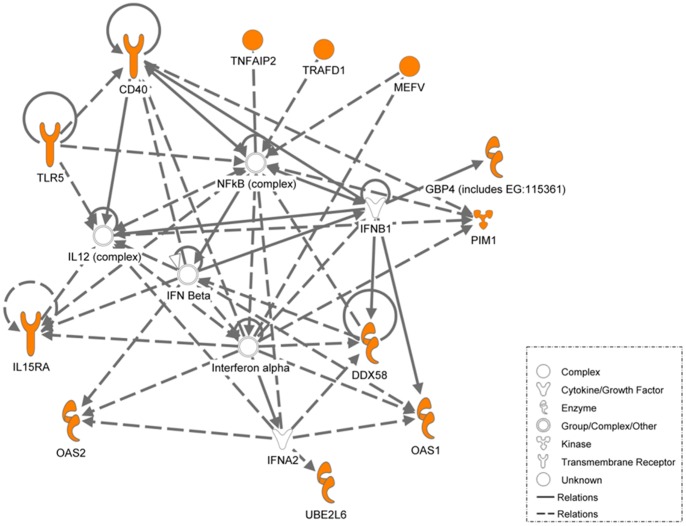
Ingenuity pathway analysis of the key genes identified. Ingenuity pathway analysis of the genes differentially regulated and common to the three systems described above (in orange). Network of genes in the interferon 1 signaling pathways that were found to be common in active TB patients, BCG-infected THP-1 cells at 20 hours and mice infected with active TB are illustrated. The lines in between genes represent known interactions, with solid lines representing direct interactions and dashed lines representing indirect interactions.

We then proceeded to validate the microarray findings for these 26 genes using quantitative RT-PCR (qRT-PCR); for successful validation, the direction for the fold-change in gene expression using qRT-PCR should be consistent with the micro-array findings. A total of 17 out of the 26 genes were successfully validated via qRT-PCR in the longitudinal patient cohort and 15 out of the 17 genes were validated in the human macrophage model (BCG-infected THP-1 cells) (**Table 6**). These 15 genes were common to both the human models (longitudinal patient cohort and BCG-infected THP-1 cells). However, only 5 out of the 17 genes could be validated in the *in vivo* mouse model (**Table 6**). Considering all validation data, 3 genes (*IL15RA*, *UBE2L6 and GBP4*) out of the 26 genes common to all 3 models were found to be consistently and reproducibly upregulated across 3 model systems (**Figure 6** **)**. It is clear from the network analysis (**Figure 7**) that *IL15RA*, *UBE2L6* and *GBP4* are involved in the type-I interferon signaling response.

## Discussion

Comparing gene expression profiles in 3 models of TB, a longitudinal human TB cohort study comprising of newly-diagnosed TB patients followed up through treatment, a BCG-infected human macrophage cell line and an *in vivo* mouse lung TB infection model, we identified in all three models a highly prominent type-I interferon molecular signature. This result significantly extends those of Berry et al [15], and Maertzdorf et al [16]. who found highly similar signatures in the blood of TB patients in Europe and South Africa; our results now confirm and extend this to a TB endemic setting on a third continent in a different host genetic as well as TB epidemiological setting, namely Indonesia. In addition, our results confirm and validate part of the findings in two independent models of TB infection, thus connecting the TB patient blood expression profiles to pulmonary and macrophage expression signatures.

Our un-biased whole genome microarray analysis revealed that the most prominent host responses in all three models of infection were immune related. The most significant pathways were interferon mediated immunity, macrophage mediated immunity and cytokine and chemokine mediated signaling pathways. Further analysis of the differentially expressed genes in the TB patient study revealed that the type-1 interferon signaling activity was the predominant pathway up-regulated during active disease, and that there was concurrent down-regulation of interferon-gamma. This is consistent with findings that key protective immune mechanisms are repressed by type-1 interferon signaling during active TB infection [21], [22] as a possible immune evasion response.

The network analysis of genes found to be commonly regulated in all 3 independent TB disease models showed that the most significant network was mainly involved in IL-12, IFN-α and IFN-β signaling. This is indeed noteworthy as evidence implicating the involvement of type 1 interferon (IFN-α and IFN-β) in TB have only begun to emerge very recently [15]. In the present study, we have shown an up regulation in type 1 interferon (α/β) and the genes involved. Kuchtey *et al*., showed that during early infection (2–3 weeks post infection) there was an enhanced growth of *M. bovis* BCG in the lungs of mice deficient in IFN-αβ receptors, and during late infection the burden of BCG was similar in lungs of IFN-αβ^−/−^ and wild type mice [23]. Desvignes *et al*. recently reported a similar non redundant protective role for type-1 interferons during early *M.tb* infection in a mouse model [24]. In a large human study using whole blood from active TB patients *vs.* uninfected controls, Berry *et al*., found an increase in type 1 IFN which was attributed predominantly to neutrophils [15]. Here, however, we present highly similar findings in TB patients’ blood leucocytes, which were depleted of neutrophils through Ficoll density centrifugation, but still include monocyte/macrophages. The fact that the latter are the most likely source of this signature was supported by the finding of a similar signature in an independent, human macrophage cell line model of mycobacterial infection. Also in another study using whole blood to compare gene expression profiles in TB patients with active disease *vs.* individuals with latent *M.tb* infection several interferon inducible genes were found to be profoundly upregulated, including IFN-α protein 6 and 27 and ISG15 [12]. Furthermore, exogenously added IFN-α was found to inhibit the priming effect of IFN-γ on key cytokine release by monocytes [22]. IFN-β was found to have a similar effect [25]. This is compatible with a model in which enhanced type I interferon signaling inhibits type II interferon mediated protective immunity in TB.

It is unsurprising that of all immune cells and corresponding signaling systems (T-cells, B-cells, monocytes/dendritic cells/macrophages, natural killer cells), the macrophage-mediated immunity process was consistently found to play a dominant role (**Tables 1** **–** **4**) in the transcriptional response in each of the three different study models. This highlights the key role that *M.tb*/macrophage interactions play during infection. Alveolar macrophages phagocytose inhaled *M.tb* by a wide range of receptors such as complement receptors CR1, 3 and 4; mannose receptors; Toll-like receptors; C-type-2 lectin receptors and scavenger receptors [26]. The further influx of macrophages following the formation of a primary lesion further highlights the key role of these professional phagocytes in pulmonary host defence in TB [12]. *M.tb* however inhibits macrophage activity by inhibiting autophagy, an important cellular mechanism to control intracellular *M.tb* [27], [28]. In addition, *M.tb* blocks the maturation of phagosomes such that they fail to traffic further along the endosomal-lysosomal pathway and fail to fuse with lysosomes. One newly identified receptor which may be important in this process is human TLR8. A polymorphism resulting in a missense mutation in TLR8 (Met1Val) was recently shown to be strongly associated with human susceptibility to TB [29]. Increased credence to the possible involvement of TLR8 in upstream *M.tb* recognition is lent by findings from the expression array in the longitudinal patient cohort, which highlighted the significance of the immunity and defense pathway in active disease, including TLRs. It may be of interest to note that TLR8 is absent from mice, which could account for part of the differences in TB infection biology between humans and mice.

Further validation of our findings using qRT-PCR revealed that *IL15RA*, *UBE2L6* and *GBP4* were involved in this molecular signature in all three models (**Figure 6**). *IL15RA* encodes for interleukin-15 (IL-15) receptor alpha (IL-15Rα), a common cytokine receptor expressed by macrophages that plays an important role in the development, survival, and proliferation of various immune cells (e.g. natural killer and CD8^+^ T cells). IL-15, which is co expressed with IL-15Rα by antigen presenting cells allowing trans-production of cytokines to immune effector cells [30] is generally considered to be a regulator of T cell homeostasis as it cooperates with other cytokines like IL-2 and IL-7 to maintain pools of naive and memory T cell populations. Mortier *et al*., has described that IL-15Rα expression on macrophages supports the early transition of antigen specific effector CD8^+^ T cells to memory cells [31]. As these processes are critical in the early immune response against microbial infections, and as data on the potential protective effects of IL-15 on murine experimental tuberculosis is starting to emerge, further studies are necessary in order to document the precise mechanism of action of IL-15-mediated type-I interferon signaling on *M.tb* survival and proliferation.

The second gene with consistent validation across the three TB models studied here, *UBE2L6*, is a member of the ubiquitin family of proteins. The modification of proteins with ubiquitin is an important cellular mechanism for targeting abnormal or short-lived proteins for degradation, and recent work linking the ubiquitin family of proteins to type-I interferon mediated host immune responses is now beginning to emerge [32]–[35].

The third gene, *GBP4,* encodes Guanylate binding protein 4. With IFN-α being a main hypothesis in our study, it is reassuring that *GBP4* was commonly found in all 3 models, and indeed GBP4 displays a direct interaction with IFN-α (**Figure 7**). Type 1 and 2 IFNs have been shown to differentially regulate the *GBP* family of genes, and GBP4 is exclusively regulated by IFN-α production through macrophages [36].

In our study a substantial number of overlapping genes were identified when comparing BCG infected human macrophage cells and circulating leucocytes from TB patients with active disease (**Figure 3**). The majority of these genes was present in the immune-mediated and host defense pathways. It needs to be highlighted that these genes were also common when the mouse TB infection model was compared to the data from the human models (**Figure 4**). The genes overlapping in all three systems were further validated with qRT-PCR. The 15 genes that were successfully validated in the THP-1 cell line model by qRT-PCR completely overlapped with the 17 genes validated by qRT-PCR in the longitudinal patient cohort. (**Figure** **6**). This is not surprising since both models share a common human host background. The fact that only 3 genes were common to all three models may reflect important differences between the human and mouse models of TB. The PCR validation of these genes may represent an underestimate of the of the true array findings as the detection probes did not directly overlap with the array probes and so may not be able to confirm the specific RNA transcript identified by the array, particularly in the mouse to human comparisons. Although the use of BCG rather than *M.tb* in the THP-1 infection model may also have contributed to this relatively low number, it should be emphasized that the analysis focused only on those genes found in all three models, and that the variation between the *M.tb* infected human patients and the *M.tb* infected mice was significantly larger that that between the *M.tb* infected human patients and the BCG infected THP-1 cells. Our approach thus excludes the identification of genes based on only one individual model, and identifies genes only when they have been validated in three models, which is a significant advance compared to previous studies. In our study the mice were infected with *M.tb* intravenously, and it will be of interest to see whether infection via the aerosolic route, which is reflective of natural TB transmission, would have yielded similar response patterns. A cardinal feature of human TB is of central caseous necrosis in pulmonary granulomatous lesions, while such necrotic lesions are not seen in the mouse TB infected lung. Caseous necrosis is considered an essential step towards developing cavernous TB, in which phase *M.tb* bacilli are released into the open airways prior to transmission. Thus, there are clear differences in the pathological manifestations of TB in human and mouse systems and this difference in disease pathology has been central to the argument that the mouse is an imperfect model for TB. However, notwithstanding these differences, we were able to observe consistent molecular signatures of active TB across the distinct models, thus supporting the general conclusion that mouse models are appropriate for studying certain aspects of the immune molecular host response to infection.

The use of TB patients cohorts, *in vitro* human macrophage cell lines and inbred mouse models of TB have provided new information which is in line with, but also significantly extends recent work from other groups [15], [16]. Collectively, these studies demonstrate the major role of dysregulated inflammation in general, and of strongly enhanced type 1 IFN-αβ signaling in active TB disease. Importantly whereas this latter finding was originally attributed to the activity of neutrophils, we here find these same gene expression patterns also in cells devoid of neutrophils, such as PBMCs and macrophages. Finally, our study discovered genes commonly expressed in all three TB models we have studied, for example *IL15RA*, *UBE2L6* and *GBP4* which are all common to the IFN-α network. Future studies should extend this also to protein expression profiles to determine whether these differences are also present at the protein level. This will allow further insights into their possible function during TB disease and *M.tb* infection, and help to better delineate new TB biomarker signatures.

## Materials and Methods

### Ethical Statement

This study was approved by the Ethical Committee of the Faculty of Medicine, University of Indonesia, Jakarta, and by the Eijkman Institute Research Ethics Committee, Jakarta, and written consent was voluntarily signed by all patients and control subjects [37].

### Patient Recruitment

After informed consent, 23 new pulmonary tuberculosis patients above 15 years of age were recruited from an outpatient tuberculosis clinic in central Jakarta (Indonesia) [37]. Diagnosis of TB was performed according to the World Health Organization criteria, on the basis of the clinical presentation and a chest X-ray radiograph (CXR) and confirmed by sputum microscopy positive for mycobacteria using Ziehl-Nielsen stain. Free anti-TB drug treatment was provided to all patients and consisted of a standard regimen of isoniazid, rifampin, pyrazinamide and ethambutol (2HRZE, 4H3R3) for a total of 6 months according to the national TB program. Treatment was supervised once weekly by a directly observed treatment program.

Human immunodeficiency virus (HIV)-seropositive patients, diabetes mellitus affected patients, patients with heart diseases and patients with incomplete data records were excluded. 23 randomly selected control subjects with the same sex and age (+/−10%) were recruited from neighboring households, with first degree relatives excluded. Controls with signs, symptoms and CXR results suggestive of active TB, a history of prior anti-TB treatment, diabetes mellitus or incomplete data entry were excluded. HIV status was not tested in control group. Indonesia is classified as a country with a low HIV prevalence of ≤0.1% during the sample period [38]. Patients’ self reported ethnicities were recorded upon recruitment. A Javanese origin characterized three groups – the Jawa, Betawi and Sunda – and altogether comprised more than 80% of the total sample. The non-Javanese category included individuals born on other Indonesian islands.

### RNA Extraction

Blood samples at three time points of disease spectrum (active phase: 0 weeks, treatment phase: 8 weeks and convalescent phase: 28 weeks) were obtained and collected in heparinized tubes. Peripheral blood mononuclear cells (PBMCs) were separated using a Ficoll density gradient, and kept frozen until use. Total RNA was extracted using the RNeasy Mini Kit (Qiagen, Germany) according to the manufacturer’s instructions, and subsequently subjected to DNAse treatment using RNAse free DNAse Set (Qiagen, Germany). RNA integrity was assessed by 1% agarose gel electrophoresis and samples of poor quality were excluded from analysis.

### *Mycobacterium bovis* Bacillus Calmette Guérin (BCG) Infection of Differentiated THP-1 Macrophages

The human monocytic cell line THP-1 (ATCC, Rockville, MD) cells were cultured in RPMI 1640 supplemented with 10% FBS, penicillin (100 U/ml) and streptomycin (100 µg/ml) (Invitrogen). Cells were plated at a density of 10^6^ in 6-well tissue culture plates (Nunclon). Monocytes were allowed to adhere and differentiate into macrophages for 48 hours with 5 nM PMA (Sigma Aldrich) at 37°C in a humidified atmosphere of 5% CO_2_. Differentiated macrophages were infected with BCG at an MOI of 5∶1 and incubated at 37°C, 5% CO_2_. Infected cells at 4 hr post infection were washed twice with RPMI without antibiotics to remove un-ingested and un-adhered bacteria to minimize cell death and re-incubated for a further 16 hours. The cells were washed once in 1× distilled PBS before 350 µL RTL buffer was added to lyse the cells and RNA extraction was then carried out using the RNeasy Mini Kit (Qiagen, Germany), according to the manufacturer’s instructions.

### C57BL/6 Mice Infected with Acute TB

C57BL/6Jbrc female mice (18–19 weeks old) were purchased from the Medical Research Centre (Biopolis, Singapore) from the specific pathogen free (SPF) facility. Mice were kept at the Novartis Institute for Tropical Diseases (NITD, Singapore) ABSL-3 facility for a week before the experiment. The experiment was approved by the Institute Animal Care and Use Committee of NITD. A total of eleven mice were used. Five mice were infected intravenously with 0.2 ml (approximately 10^6^ CFU) of *M.tb* Erdman strain. Six mice served as mock infected controls.

Briefly, *M.tb* Erdman strain was grown in Dubos broth to the logarithmic stage. Bacteria were diluted in saline containing 0.05% Tween 80 for infection in mice. Mock infected mice were intravenously administered PBS containing 0.05% Tween 80. After four weeks, infected and control mice were sacrificed. Half of the lungs was immersed in saline containing 0.05% Tween 80 for homogenizing to determine the CFU (data not shown) and the other half was immersed in the RNA later® stabilizer solution (Ambion®, USA) prior to preparing tissue lysate for extracting total RNA.

### RNA Extraction from Mice Lungs

For preparation of tissue lysate, lung tissue immersed in the RNA later® stabilizer solution (Ambion®, USA) was transferred to the lysing Matrix D tube of FastPrep system Pro Green Kit (Plants and Animals) (Qbiogen, Germany). Tissue lysate was then prepared according to the manufacturer’s instructions.

Total RNA was extracted from tissue lysate using the RNA Easy kit (Qiagen, Germany). Extracted RNA was subjected to DNAse treatment using RNAse free DNAse Set (Qiagen, Germany). Integrity of the RNA was assessed by 1% agarose gel electrophoresis and samples of poor quality were excluded from analysis.

### Microarrays

Total RNA (250 ng) was amplified in a single round of *in vitro* transcription amplification that allowed incorporation of biotin-labelled nucleotides using the Illumina TotalPrep RNA amplification kit (Ambion, Austin, TX), according to the manufacturer’s instructions. cRNA (750 ng) of each sample was hybridized to an Illumina HumanRef-8 V3 BeadChip (containing probes to 24526 RefSeq gene sequences) at 58°C for 16 hours according to the manufacturer’s instructions (Illumina, Inc., San Diego, CA). This was followed by washing, blocking and streptavidin-Cy3 staining steps, followed by scanning with a high resolution Illumina bead array reader confocal scanner, according to the manufacturer’s instructions. For all arrays at the different time points, a rigorous quality check was carried out to ensure good performance before the array data was extracted. The data extraction was performed by using Illumina BeadStudio v3 software and uploaded into Genespring GX10 (Silicon Genetics, Redwood City, CA) software for downstream analysis.

### Array Normalization

The standard normalization procedures recommended by Genespring software for one-colour array were followed. Data transformation was corrected for a low signal, with values recorded at <0.01 increased to the minimum (0.01). Chip variability was accounted for using per-chip normalization by dividing all of the measurements on each chip by a 75^th^ percentile value. Probe set variability for different genes were accounted for using per-gene normalization by dividing all genes by the median of all genes. Analysis was restricted to probe sets for which a detection level of 0.9899 was obtained in at least 50% of arrays in at least one condition stated. Following quality control of probes, statistical analysis, hierarchical clustering and functional classification were performed.

### Selection of Differentially Expressed Genes from Microarray Data

Differentially expressed genes were selected from the normalized data by using a procedure known as significance analysis of microarrays (SAM) [39] as installed on Genespring GX10. The statistic in SAM is given as d = (u_1_−u_2_)/(s−s_0_), where the numerator is the group mean difference, s is the standard error and s_0_ is a regularizing constant. Setting s_0_ = 0 will yield a t-statistic. This value, called the fudge constant, is found by removing the trend in d as a function of s in moving windows across the data to reduce false positive results. Since the statistic is not t-distributed, significance is computed using a permutation test. Genes with computed statistic larger than the threshold are called significant. 1000 permutations were conducted and a threshold false discovery rate (FDR) of 0.05 set to determine the differentially expressed genes which were significant, and subsequently subjected to filtering through fold change of 2 or greater.

Clustering using Self Organizing Map (SOM) function was used to group genes with different gene expression profiles, with Euclidean distance metric, a maximum iterations of 50, grid rows of 3 by 4, initial learning rate of 0.03, initial neighborhood radius of 5, neighborhood bubble type, with a hexagonal grid topology.

### Gene Ontology Clustering

The PANTHER (www.pantherdb.org) online gene expression analysis system was used to group significantly overrepresented genes into functional relationships. Similarly, the Ingenuity Pathway Analysis (IPA; www.ingenuity.com) was also used to classify differentially expressed genes into functional relationships and further show canonical pathways and networks involving these genes, with nodes defining potentially critical host mediators of infection. The canonical pathways are described in the library of the Ingenuity Pathways knowledge base and are based on well-known established textbook literature where molecular links are known. The networks are built from a compilation of literature searches. Connecting lines between nodes represent one or more publications which imply a direct or indirect connection between the genes. The Ingenuity program is updated monthly to reflect new publications.

For canonical pathways, the significance of the association was measured in two ways: (i) by the ratio of the number of genes from the data set that map to the pathway divided by the total number of genes in that pathway and (ii) by using the Benjamin-Hochberg multiple corrected p-value to obtain a p-value determining the probability that the association between the genes in the data set and the canonical pathway is explained by chance alone, correcting for multiple testing of the same genes against different canonical pathways.

Network-wise, IPA computes a score for each network related to the fit of the input set of differentially expressed genes. The score is obtained from the likelihood that the focus genes in a network are together due to chance alone, with a score of 2 being equivalent to a p-value of 0.01.

### Quantitative RT-PCR (qRT-PCR) by Fluidigm System

Using the extracted RNA from the three systems above, 100 ng of total RNA was reverse transcribed using the High-Capacity cDNA Kit for 1000 reactions (ABI, USA), and processed for the 48.48 Fluidigm Microfluidic Chips, according to the manufacturer’s instructions, together with data analysis using the Biomark qRT-PCR analysis and Biomark Melting Curve analysis software (Fluidigm, USA). We normalized the qRT-PCR data in relation to a reference gene expression quantitation using the gene 18S, according to well established procedures. The RNA samples used for PCR were the same as those used for the microarray.

### Microarray Statistical Analysis

For microarray analysis, the Illumina HumanRef-8 V3 BeadChip was utilized, containing more than 24,000 genes. For the initial analysis, the T-test with Benjamin-Hochberg procedure was used to determine differentially expressed genes from the normalized data in any one condition from the controls (see Materials and Methods). Analysis of variance (ANOVA) was used to determine differentially expressed genes across patient groups.
